# Flexor hallucis longus tendon morphology in dancers clinically diagnosed with tendinopathy

**DOI:** 10.1007/s40477-023-00793-5

**Published:** 2023-06-25

**Authors:** Pamela Mikkelsen, Alyssa Andersen, Hai-Jung Steffi Shih, K. Michael Rowley, Kornelia Kulig

**Affiliations:** 1 Division of Biokinesiology and Physical Therapy, University of Southern California, 1540 Alcazar St #155, Los Angeles, CA 90033 USA; 2 Department of Biobehavioral Sciences, Teachers College, Columbia University, New York, NY USA; 3 Kinesiology Department, California State University East Bay, Hayward, CA USA

**Keywords:** Ultrasound, Tendon structure, Tendinopathy, Adaptation, Flexor hallucis longus, Dance

## Abstract

**Purpose:**

The unique demands of dance technique make dancers more prone to certain pathologies especially of the foot and ankle. Flexor hallucis longus (FHL) tendinopathy, colloquially known as “dancer’s tendinopathy,” is common in dancers and not well studied. The purpose of this study was to assess if morphological alterations in tendon structure occur as an adaptive response to dance activity by comparing the FHL tendon in dancers to non-dancers, and if pathology further alters tendon morphology in dancers clinically diagnosed with tendinopathy.

**Methods:**

Three groups of ten participants were recruited (healthy non-dancers, healthy dancers, and dancers with FHL tendinopathy). Ultrasound images of the FHL tendons were analyzed for macromorphology by measuring the tendon thickness. The micromorphology was analyzed by determining the peak spatial frequency radius of the tendon. Our study did find increased tendon proper and composite tendon thickness in dancers with tendinopathy but no difference between asymptomatic dancers and non-dancers.

**Results:**

There was no significant difference in micromorphology found between any of the groups. As expected, dancers with tendinopathy demonstrated increased composite tendon and tendon proper thickness however, there was no evidence of adaptive thickening of the FHL tendon as might be expected for the dance population. There was also no evidence of micromorphological changes in the presence of clinically diagnosed FHL tendinopathy.

**Conclusion:**

Because of the limited normative data for this pathology, these results can help improve diagnosis and therefore treatment for dancers to decrease the impact of this injury on their careers.

## Introduction

The athletic art of dance imparts unique mechanical demands on the musculoskeletal system with its requirements of remarkable flexibility, power, and precision of human movement. For many dance styles, especially classical ballet, much of this demand is centered around the lower extremity and particularly the foot and ankle [1, 2]. A common position for dancers is a heel rise standing either on the balls of the feet or tips of the toes (*demi* and full *pointe*, respectively). In these positions, the Achilles, posterior tibial, peroneal, and flexor hallucis longus (FHL) tendons create a mechanical stirrup to actively stabilize the foot and ankle [3]. The triceps surae allows the dancer to achieve end-range ankle plantar flexion and the posterior tibial and peroneal tendons act to stabilize the ankle in the frontal plane. The FHL works as a major stabilizer with demands of additional elongation due to first toe dorsiflexion in *demi pointe* and active first toe plantarflexion in full *pointe* positions. In addition to static poses, dancers also perform dynamic movements like jumps and leaps which places increased demands on the foot and ankle [1, 2, 4]. Combined, these static poses and movements are thought to contribute to the high prevalence of overuse injuries in dancers and especially to tendinopathies of the foot and ankle [5–7].

The particular aesthetic demands of dance make FHL tendinopathy one of the most prevalent of all lower extremity tendinopathies reported in female dancers [7–11]. It is even colloquially known as “dancer’s tendinopathy.” Clinically, FHL tendinopathy can present as pain in the posteromedial ankle with weightbearing ankle dorsiflexion or heel rise position, pain with palpation along the tendon, limited range of motion of the ankle and toe, and pain with resisted first toe flexion. The treatment can range from decreased activity, conservative management, and surgery [8, 9]. The FHL tendon is enclosed in a sheath and passes behind the medial malleolus, under the sustentaculum tali of the calcaneus, and through the sesamoid bones on its way to the first toe. Constriction at these areas can contribute to inflammation of the synovial membrane in the tendon sheath with an accumulation of synovial fluid. Altered mechanical function also occurs due to the development of nodules and alterations to the tendon proper and, clinically, this can present as trigger toe in advanced disease process [12]. The presentation of FHL tendinopathy may differ from more extensively studied tendinopathies like Achilles or patellar due to the presence of this sheath, making it structurally more similar to the posterior tibial tendon [13]. The differences in how tendon pathology develops in a tendon covered in paratenon like a patellar tendon compared to a sheathed tendon like posterior tibial tendons limit direct comparisons to commonly studied tendons when investigating tendinopathy of these sheathed tendons.

The body adapts based on the forces it experiences, and tendon adaptations can be seen on macro- and micromorphologic levels. Tendons demonstrate thickening as a normal adaptive mechanism with increased use, but with unhealthy loads, there can be maladaptation. This can be seen as pathologic thickening of the tendon itself or swelling of the structures surrounding the tendon [13]. In the presence of tendinopathy, there are micromorphological changes seen in the tissue from a dysfunctional healing response, and the tendon presents with disrupted or thinner collagen bundles, hypercellularity, an increase in proteoglycans, and often neovascularization [14, 15]. These tendon adaptations are further associated with altered mechanical properties and decreased stiffness of the tendon [14, 16].

Ultrasound has been used to characterize changes in macromorphology by looking at cross-sectional area or diameter to assess tendon thickness. Increases in tendon thickness have been shown in tendons like the patellar tendon or Achilles tendon for athletes that have normal adaptive responses as well as symptomatic tendinopathy [17, 18]. Ultrasound has also been used to assess micromorphology and the presence of tendinopathy by assessing tissue structure and collagen fiber orientation. Qualitatively, pathology can be identified as areas of hypoechoicity, and quantitatively, the organizational structure of ultrasonic images using peak spatial frequency radius has been used to objectively identify tendinopathy [19].

Little research has been conducted on FHL tendinopathy especially in the dance population resulting in limited normative data. There are no studies that specifically characterize FHL tendon morphology in dancers with and without pathology nor are there any studies comparing this to non-dancers. This makes determining the difference between healthy and unhealthy tendon changes challenging. Without this knowledge, practitioners are limited in their ability to diagnose, treat, and prevent progression of FHL tendinopathy in dancers. Thus, the purpose of this study was to assess if morphological alterations in tendon structure occur as an adaptive response to dance activity by comparing the FHL tendon in dancers to non-dancers, and if pathology further alters tendon morphology in dancers clinically diagnosed with tendinopathy.

## Methods

### Participants

Three groups of participants were recruited: dancers with FHL tendinopathy (*n = *10), healthy dancers (*n = *10), and non-dancers (*n = *10). Recruitment occurred prospectively from collegiate dance and non-dance programs, professional dance companies, and from within the University of Southern California Division of Biokinesiology and Physical Therapy. Data was acquired from a larger biomechanical study [20]. The study was done with approval from the institutional review board of the University of Southern California, Los Angeles, CA, and informed consent was acquired from all participants. The participants were screened for inclusion based on the criteria below.

All participants were between the ages of 18 and 45 to minimize the effect of age-related changes to tendon morphology. Dancers were included if they had at least 10 years of formal dance training and were currently dancing at least 3 hours per week. Non-dancers were included if they had no history of formal dance training.

Dancers with suspected FHL tendinopathy were screened by a physical therapist specializing in the management of dance injury. Clinical differential diagnosis included history of symptoms and a condition-specific physical exam. Participants were also screened for other causes of posterior ankle pain using this clinical examination as well as ultrasound imaging of the Achilles to rule out Achilles tendinopathy. Dancers with clinically confirmed FHL tendinopathy were included if they reported that the posterior ankle pain had limited their ability to train within the last three months. They were excluded if they demonstrated signs or symptoms of concurrent Achilles tendinopathy or other pathology of the foot and ankle. They were excluded if they reported a history of surgery or lower extremity injury other than FHL tendinopathy within the last year that required time off from dancing. Healthy dancers and non-dancers were excluded if they reported any history of surgery or lower extremity injury within the last year that required them to modify their participation in physical activity.

### Examination and image acquisition procedure

Testing took place at the Jacquelin Perry Musculoskeletal Biomechanics Research Laboratory at the University of Southern California. Examination procedures were explained to each participant, and written informed consent to participate and have the data published was obtained as approved by the Institutional Review Board of the University of Southern California. All participants completed self-reported medical history and dance history questionnaires and were screened by a licensed physical therapist in order to confirm eligibility. The participants with posterior ankle pain were assessed and those that were found to have other causes for the symptoms like Achilles tendinopathy or posterior ankle impingement were eliminated from the study. The tests and measures that were used to indicate FHL tendinopathy included reports of pain with *relevé* or heel rise and *grande plié,* pain with palpation at the knot of henry or posterior ankle, pain with resisted first toe plantarflexion, popping along the FHL tendon with first toe flexion or extension, triggering into flexion for the first toe, positive Thomasen’s sign with limited first toe dorsiflexion while in knee extension and ankle dorsiflexion [8, 21]. The dancers that were determined to be positive for FHL tendinopathy were included in the study.

**Fig. 1:**
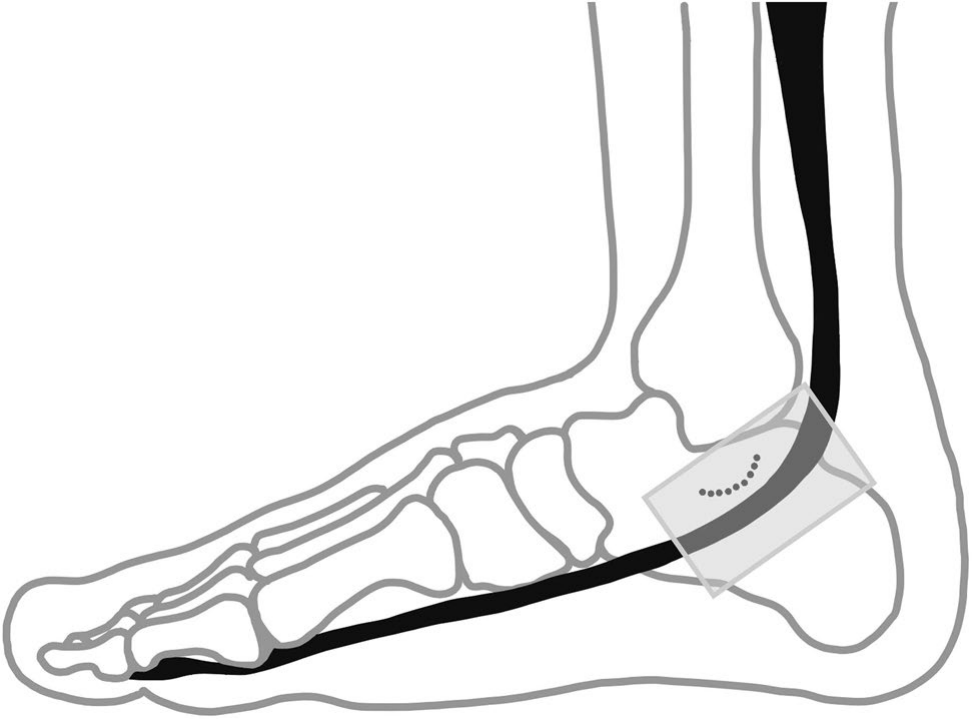
Schematic presentation of the placement of the ultrasound transducer at the medial ankle capturing the longitudinal view of the Flexor Hallucis Longus (FHL) tendon as it travels inferiorly to the sustentaculum tali of the calcaneus. Box depicts placement of the ultrasound transducer head. Dotted line delineates sustentaculum tali. Black solid line identifies the path of FHL tendon

Longitudinal B-mode ultrasound images of the FHL tendons were acquired by the same sonographer for all subjects using a commercial ultrasound scanner with linear probe (GE LOGIQe with 12L-RS linear transducer, GE Healthcare, Little Chalfont, UK). In addition, doppler mode was used when acquiring longitudinal images to identify possible intratendinous neovascularization (Fig. 1). Both limbs were scanned in dancers with FHL tendinopathy, whereas only the preferred stance limb was scanned in healthy dancers and non-dancers. In dancers with bilateral symptoms, the stance limb was the limb with more severe symptoms in all but two participants.

During image acquisition, the participant lay prone on the test table with feet hanging over the edge and ankle held in neutral by the examiner. Longitudinal images were taken parallel to the long axis of the tendons. Images of the FHL tendon were taken with the ultrasound probe placed posterior to the medial malleolus and medial to the Achilles tendon (Fig. 1). This location was further confirmed by palpation and active/passive movement of the first hallux. Static images of the FHL were obtained as well as 3 s video clips of the FHL tendon recorded during passive great toe flexion and extension.

### Image processing

The scans were digitally stored on the ultrasound machine as scanner-compressed JPEG intensity images. The image data of the were imported into ImageJ software (National Institute of Health, v.1.366, Bethesda, MD) for assessment of macromorphology and into Matlab (MathWorks, Natick, MA) for assessment of micromorphology.

### Image analysis: macromorphology

**Fig. 2:**
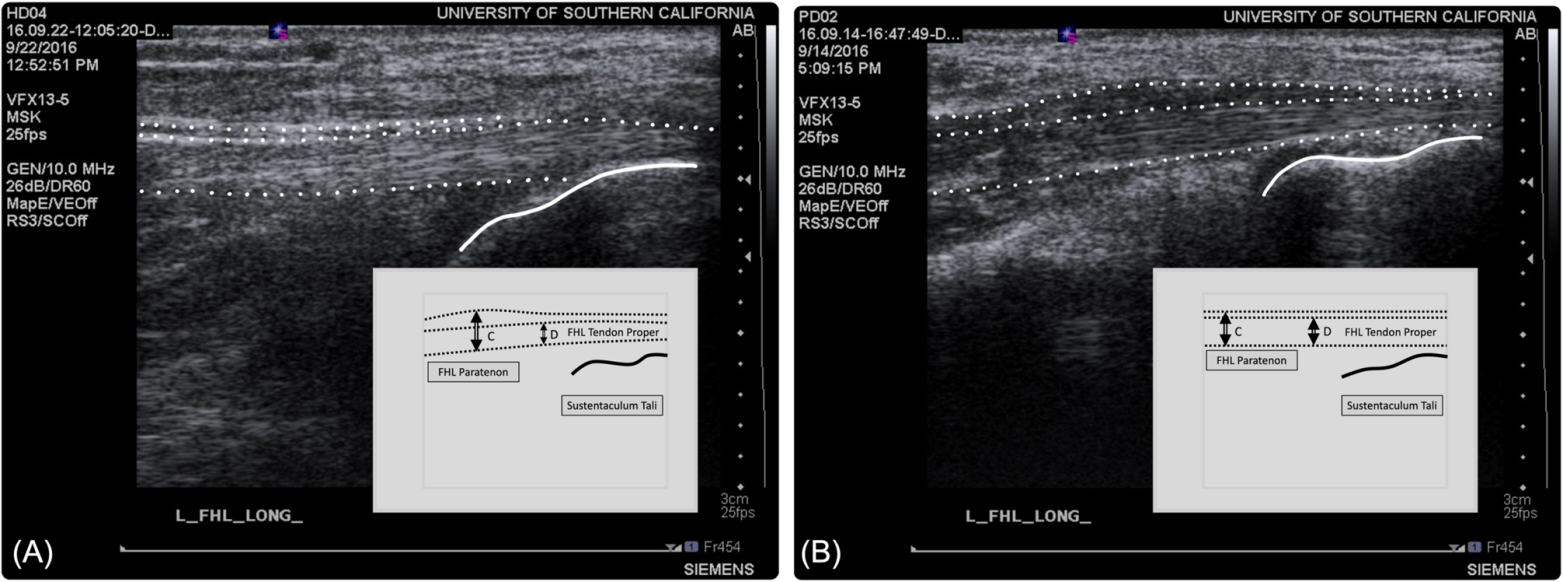
**A** B mode ultrasound image of the Flexor Hallucis Longus tendon in a representative healthy subject. **B** B mode ultrasound image of the Flexor Hallucis Longus tendon in a representative dancer with tendinopathy. Solid line illustrates sustenaculm tali. Dotted lines delineate paratendon and the tendon proper. Boxed insert schematically demonstrates longitudinal measurements of **C** FHL paratendon and **D** FHL tendon proper

Tendon thickness was measured on the longitudinal image at the thickest section of the tendon clearly visualized within the scan. Two separate measurements were recorded: the first capturing composite tendon thickness, including paratenon and tendon proper, and the second capturing tendon proper thickness alone (Fig. 2). All measurements were confirmed with the ultrasound video clips to aid in accurate delineation of tendon borders. Measurements were taken three times and then averaged.

### Image analyses: micromorphology

**Fig. 3:**
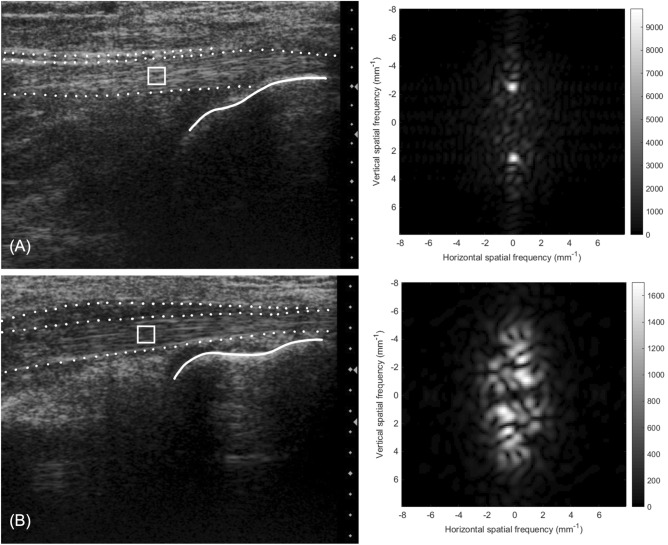
Frequency analysis of FHL tendon ultrasound images. Left: original B-mode image of FHL tendons with a sample kernel selection [white square, region of interest (ROI)] for a representative healthy dancer **A** and dancer with tendinopathy **B**. Dotted lines indicate outline of FHL tendon proper and paratenon. Right corresponding graphic representations of the frequency spectrum analysis of the selected ROI

Peak spatial frequency radius (PSFR) is defined as the distance from origin to spatial frequency peak of greatest amplitude on two-dimensional fast Fourier transformation (2-D FFT) spectrum [19]. To do this, a region of interest (ROI) was selected on the ultrasound image in the FHL tendon’s midsubstance at the clearest point visualized within the scan (Fig. 3). Multiple kernels (32 × 32 pixels in dimension, corresponding to 1.6 × 1.6 mm) were defined within each ROI and processed with a sliding window using a custom image analysis program (MATLAB, Mathworks, Natick, MA). In order to increase spatial frequency resolution, each kernel was zero-padded to 128 × 128 pixels in size. A two-dimensional high-pass filter was applied with radial frequency response to suppress the low spatial frequencies within the kernel. Within each kernel a 2-D FFT was conducted in order to perform spatial frequency analysis. PSFR, which is the spatial frequency with the largest power in the image, was extracted and averaged over each kernel of the ROI. Figure 3 illustrates a sample analysis of the tendon ultrasound images including a corresponding graphic representation of the frequency spectrum analysis of the selected ROI.

### Statistical analysis

Data were analyzed using SPSS Statistical Software (SPSS Inc, Chicago, IL) with a significance level set at *p* = 0.05. Normality of the data was tested using the Shapiro–Wilk Test. If data was determined to be normally distributed, a one-way ANOVA with Bonferroni post hoc test was used to test statistical significance between groups. If data were determined to deviate from a normal distribution, a Kruskal Wallis test with multiple comparisons was performed to assess for significance between groups. Comparisons were made between groups for demographics, micromorphology, and macromorphology of the FHL tendon.

## Results

### Demographics

There were no significant differences in age, height, or weight between dancers with tendinopathy, healthy dancers, and non-dancers (Table 1).

**Table 1:** Descriptive characteristics of study participants per group; morphometric, demographic, and years of dance training

Participant demographics			
	Healthy non-dancers	Healthy dancers	Dancers with tendinopathy
Age (years)	23.7 ± 3.3	26.5 ± 4.8	23.8 ± 5.1
Body weight (kg)	68 ± 14.8	60.7 ± 7.7	62.7 ± 4.3
Height (cm)	168.13 ± 7.74	165.45 ± 8.03	166.23 ± 3.99
Years of dance training	NA	15.2 ± 5.4	16.4 ± 3.6

### Normality

The following groups were determined to be normally distributed and thus compared using a one-way ANOVA with Bonferroni post hoc test: FHL composite tendon thickness. The following groups were determined to be not normally distributed and thus compared using a Kruskal Wallis test with multiple comparisons: FHL tendon proper thickness and FHL PSFR.

### Macromorphology

**Fig. 4:**
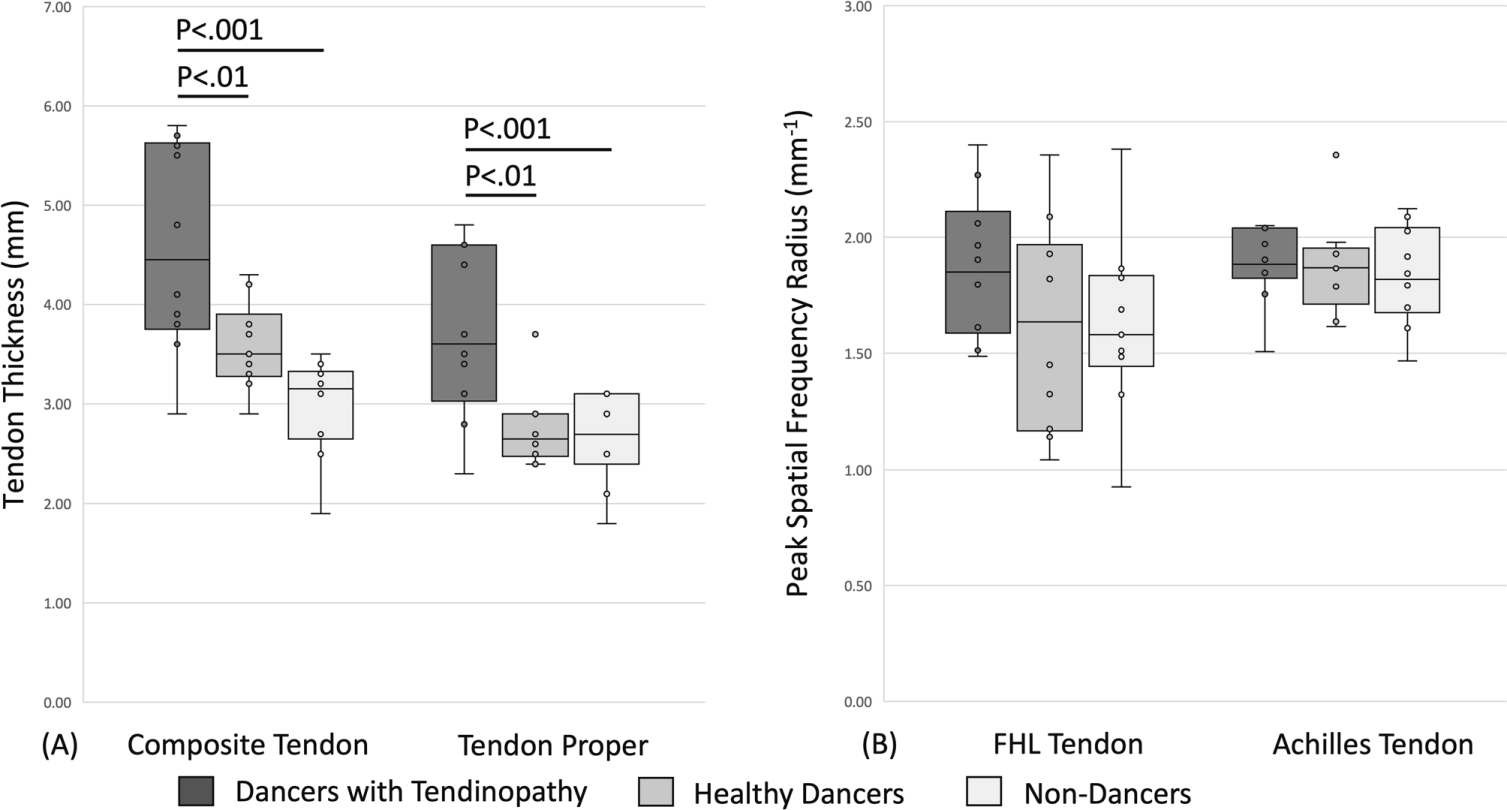
**A** Flexor Hallucis Longus tendon thickness (mm) determined from a longitudinal view of the tendon. **B** Flexor Hallucis Longus tendon and Achilles tendon peak spatial frequency radius (mm^−1^) extracted from the longitudinal view of the tendon. Standard box plots showing upper quartile, median, lower quartile, and whiskers from maximum to minimum. Each participant is represented with a circle. Solid horizontal line indicates significant difference between groups, with p value depicted above

FHL composite tendon thickness, including paratenon and tendon proper, was significantly greater in dancers with tendinopathy as compared to healthy dancers (*p* < 0.01) and non-dancers (*p* < 0.001). FHL tendon proper thickness was significantly greater in dancers with tendinopathy as compared to healthy dancers (*p* < 0.01) and non-dancers (*p* < 0.01). There was no significant difference between healthy dancers and non-dancers in FHL composite tendon thickness (*p* = 0.119) or FHL tendon proper thickness (*p* = 1.0). There was no evidence of neovascularization in the FHL tendons in any participants. (Fig. 4a).

### Micromorphology

There was no significant difference in PSFR between dancers with tendinopathy, healthy dancers, and non-dancers in PSFR of the FHL tendons (*p* = 0.229). (Fig. 4b).

## Discussion

The purpose of this study was to characterize FHL tendon macro- and micromorphology in dancers with clinically diagnosed tendinopathy and compare this to asymptomatic dancers and non-dancers. We also compared tendon morphology between asymptomatic dancers and non-dancers to determine normal tendon changes with increased use. We expected to see increased tendon thickness in dancers compared to non-dancers and further increased tendon thickness in dancers with tendinopathy compared to asymptomatic dancers and non-dancers. Our study did find increased tendon proper and composite tendon thickness in dancers with tendinopathy but no difference between asymptomatic dancers and non-dancers. There was also no significant difference in micromorphology found between any of the groups.

### Macromorphology

With respect to tendon macromorphology, tendon thickness has been shown to increase with increased loading as a natural adaptive response [22–24] as well as in the presence of tendinopathy [16, 17]. Therefore, distinguishing between normal adaptive changes and pathological changes in tendon structure can be a challenge. In addition, the bulk of current evidence comes from tendons without a sheath which may differ from sheathed tendons like the FHL. Given the increased demand on the FHL muscle and tendon during dance, we expected to see an increase in tendon thickness in all dancers compared to controls, but our results did not support this expectation, as there was no significant difference between asymptomatic dancers and non-dancers. Rowley et al. also reports no significant increase in FHL tendon thickness or cross-sectional area in a population of healthy dancers compared to healthy non-dancers [20] but Shih et al. reports a significantly increased cross-sectional area in dancers compared to non-dancers [24]. Reasons for this difference may be due to differences in data collection techniques like ultrasound probe location. Location-specific load-dependent tendon thickness increases have been shown in patellar tendons where the distal tendon demonstrates significant thickening while the mid-tendon did not [22]. Also, the location of the ultrasound probe and probe alignment may influence the cross-sectional area and sensitivity to determining differences in tendon thickness measurements.

The amount of adaptive thickening seen in asymptomatic dancers may have been influenced by participant selection. Similar to Rowley et al., the inclusion criteria for this study did not specify dance technique requirements while Shih et al. required 3 h per week of ballet training [20, 24]. Since ballet technique and especially pointe technique requires increased use of the toe flexors compared to other dance techniques, there may have been greater normal adaptive thickening of the FHL seen in this population compared to the potentially broader dance background of the participants in the current study. This may have decreased the likelihood of reaching statistical significance of FHL tendon thickness differences in dancers vs non dancers.

We chose to measure both composite tendon thickness including the synovial sheath or paratenon as well as isolated tendon proper thickness for the FHL tendons. Fluid accumulated around the tendon is attributed to inflammation and can be an indication of tendon pathology. In looking at our data, there was an increase in width seen in both the tendon proper and the composite tendon for dancers with clinically diagnosed FHL tendinopathy. This indicates that the macromorphology of the FHL tendon is altered in the presence of tendinopathy. The FHL tendon is surrounded by a synovial sheath instead of a well-adhered paratenon as seen in other non-sheathed tendons like patellar and Achilles tendons. This limits the ability to compare the process of pathology of the FHL to all tendons and instead encourages comparison with those having a similar structure like the posterior tibial tendon which is also surrounded by a synovial sheath.

### Micromorphology

Tendon degeneration and pathologic adaptation can also be assessed on a micromorphological level. Peak spatial frequency radius (PSFR), obtained via spectral analysis of the ultrasonographic images, is an indicator of tendon fiber organization. It measures the density of the collagen bundles of a tendon and a lower PSFR has been linked to tendon degeneration and suboptimal mechanical properties [17, 18, 25–27]. Micromorphological changes have been shown in the patellar tendons of athletes with clinical signs and symptoms of patellar tendinopathy [17]. In dancers with patellar tendon pain, however, there was no difference in tendon micromorphology [25]. The ages of the participants for both studies were similar but the stage of pathology for both is unclear which may account for the different findings. Regardless, this indicates a possible difference in how patellar tendinopathy presents in dancers versus sports athletes. Our study found no difference in PSFR between any of the groups. The specialized structure of the FHL tendon may also play a role in limited development of intratendinous changes since the presence of a synovial sheath may offer some protection. The posterior tibial tendon has a similar structure surrounded by a synovial sheath that demonstrates this protective pattern. In late stages of tendinopathy, lower PSFR values have been reported in non-dancer populations clinically diagnosed with posterior tibial tendinopathy only when the synovial sheath was found to be disrupted [13, 26, 28]. This supports the idea that the sheath is protective against pathologic changes in tissue organization. The lack of micromorphological changes seen in the FHL tendon in this study also supports the idea that the tendinous sheath may be protective.

### Limitations

There are limitations of this study that may help with the direction of future investigations. Inclusion criteria required dancers to have at least 10 years of formal dance training but did not specify the type of dance. As previously indicated, different dance styles impart different mechanical loads on the lower extremities and a style that does not require repeated movement from excessive ankle dorsiflexion into full ankle and toe plantarflexion may not induce the morphological adaptations to the FHL tendon like ballet and *pointe* techniques do. Specifying the requirement of ballet technique may increase the likelihood of recruiting dancers with FHL tendinopathy and highlight the unique morphological adaptations that ballet induces. The symptom severity of these dancers was also varied as the dancers presented at different stages of disease progression. We did not have any subjects with trigger toe symptoms which is an indication of higher severity of disease process. This could affect the amount of tendon adaptation seen on both a macro- and micromorphological level. This can be addressed with increased recruitment numbers and categorizing dancers into length of time with the pathology including trigger toe as inclusion criteria for more severe disease process.

Another limitation with subject recruitment is the high injury rate of dancers. Due to the demanding nature of the work, dancers often have multiple injuries at a time while performing and a majority of injures in ballet dancers are of the foot and ankle [7, 29]. While this made it challenging to recruit healthy dancers, it also made recruiting dancers with isolated FHL tendinopathy very difficult and may not accurately represent the wider population of dancers with this pathology. Including dancers with other pathologies in addition to FHL tendinopathy may increase the pool of subjects but would add complications of the data. For example, although the presence of Achilles tendinopathy was specifically excluded in the present study, there is correlation between the presence of Achilles tendinopathy and hypertrophy of the FHL muscle likely due to compensation and possibly leading to increased thickness of the FHL tendon [30].

### Clinical application

Although not the purpose of this study, these findings support using ultrasound to quantify tendon thickening to better diagnose FHL tendinopathy in a clinical setting. As we continue to gather normative data sets, tendon measurements from patients can be compared to these for a more accurate diagnosis. In the meantime, measurements within a patient could be compared bilaterally to identify potential thickening. This could be especially helpful if there are multiple injuries or pathologies suspected at the same time in the foot and ankle as is often seen in this population. Our findings do not support using ultrasound to assess micromorphology as an indication of pathology at this time. As discussed earlier, further studies are needed to look at dancers in later stages of the disease process to assess if micromorphology does change as seen in late-stage posterior tibial tendinopathy. In a population where dancers’ livelihoods depend on the health of their bodies, having an accurate diagnosis would help to direct treatment and could improve their likelihood of returning to performing without limitations.

## Conclusion

Our findings showed increases in FHL composite tendon and tendon proper thickness in dancers with tendinopathy compared to healthy dancers and non-dancers. However, there was no difference in thickness between healthy dancers and non-dancers indicating that there was no load-based adaptive thickening of this tendon. There was no significant difference found in micromorphology between dancers with tendinopathy or non-dancers indicating no change in tissue organizational structure of the tendon with clinically diagnosed pathology.

## Data Availability

The data that support the findings of this study are available on request from the corresponding author, PM, upon reasonable request. The data are not publicly available due to containing information that could potentially compromise research participant privacy.
